# The Relationship Between Anthropometric Indices of Obesity and Sleep Disturbances in Young Adults

**DOI:** 10.7759/cureus.71178

**Published:** 2024-10-09

**Authors:** Ramya K, Mukundan A

**Affiliations:** 1 Physiology, Sri Lakshmi Narayana Institute of Medical Sciences, Bharath Institute of Higher Education and Research, Pondicherry, IND; 2 Pediatrics and Child Health, Rajiv Gandhi Government Women and Children Hospital, Pondicherry, IND

**Keywords:** bmi, body composition, obesity, pittsburgh sleep quality index, sleep quality, waist circumference, waist-to-hip ratio, young adults

## Abstract

Background: Sleep is vital for maintaining overall health and influencing various physiological and psychological processes. Disruptions in sleep quality are linked to adverse health outcomes, including obesity and cardiovascular disease, particularly among young adults who are often exposed to stress and lifestyle changes. This study investigates the relationship between anthropometric indices of obesity measured through body mass index (BMI), waist-to-hip ratio (WHR), and waist circumference (WC) and sleep disturbances in young adults.

Methods: A cross-sectional study was conducted with 243 participants aged 18-30 years. Anthropometric measurements (BMI, WHR, and WC) were obtained, and sleep quality was assessed using the Pittsburgh Sleep Quality Index (PSQI). Correlation and regression analyses were performed to evaluate the relationships between anthropometric indices and PSQI scores.

Results: Higher BMI, WHR, and WC were significantly associated with poorer sleep quality, as indicated by positive correlations with PSQI scores (BMI: r = 0.28, p = 0.004; WHR: r = 0.23, p = 0.013; WC: r = 0.30, p = 0.001). Regression analysis confirmed that increases in these indices predicted worse PSQI scores, with β-values of 0.31 for BMI (p = 0.0007), 2.05 for WHR (p = 0.0003), and 0.11 for WC (p < 0.0001).

Conclusion: The study underscores a significant association between increased body and abdominal fat with poor sleep quality. The findings suggest that managing body weight and reducing abdominal obesity could improve sleep health. Future research should explore these relationships using longitudinal designs and objective sleep measures.

## Introduction

Sleep is a critical component of overall health, influencing a wide range of physiological and psychological processes. Adequate sleep is essential for maintaining metabolic homeostasis, cognitive function, and emotional well-being. However, disturbances in sleep quality and sleep patterns can lead to various adverse health outcomes, including obesity, cardiovascular diseases, and impaired cognitive function. The impact of poor sleep on health is particularly concerning among young adults, a demographic often experiencing significant life changes, such as transitioning through university education or entering the workforce. These transitions can contribute to increased stress, irregular schedules, and lifestyle changes, all of which predispose young adults to sleep disturbances [1].

Concurrently, obesity rates have been rising among young adults, making it imperative to explore the potential connections between sleep quality and body composition in this group. Recent studies have highlighted that sleep disturbances are not only common in young adults but are also associated with an increased risk of obesity and related metabolic disorders [2]. This relationship has prompted further investigation into how specific anthropometric indices, such as body mass index (BMI), waist-to-hip ratio (WHR), and waist circumference (WC), correlate with different aspects of sleep quality.

Anthropometric indices serve as valuable tools for assessing body composition and fat distribution, providing insights into an individual’s risk for obesity-related health conditions. BMI is widely used to categorize individuals based on their weight relative to height, while WHR and WC offer additional measures of fat distribution, particularly abdominal obesity, which is closely linked to metabolic risk factors [3]. Previous research has established a general association between obesity and poor sleep quality, suggesting that increased body fat, particularly in the abdominal region, may contribute to sleep disturbances through mechanisms such as obstructive sleep apnea and reduced sleep efficiency [4].

This study aims to investigate the relationship between anthropometric indices and sleep disturbances in young adults, providing insights that could help in identifying individuals at risk for sleep disturbances based on their body composition.

## Materials and methods

Study design

This cross-sectional study was conducted to investigate the relationship between anthropometric indices and sleep disturbances among young adults. The study included 243 participants aged 18-30 years. The sample size was calculated using Cochran's formula [5], and participants were recruited from volunteers at Sri Lakshmi Narayana Institute of Medical Sciences. Ethical approval for the study was obtained from the Institutional Ethical Committee. Informed consent was obtained from all participants prior to their involvement in the study, emphasizing the voluntary nature of participation and the confidentiality of their data.

This study included healthy young adults aged 18 to 30 years with regular sleep patterns, who provided informed consent and were willing to participate. They underwent anthropometric measurements and completed sleep quality assessments. Exclusion criteria included individuals with chronic illnesses, pregnancy, use of medications affecting sleep or weight, diagnosed sleep disorders, substance abuse, irregular work schedules, or any other significant health conditions that could influence sleep.

Data collection

Anthropometric Measurements

Anthropometric data were collected by using standardized procedures to ensure accuracy and reliability. Body mass index (BMI) was calculated using the Quetelet formula:

\[BMI = \frac{\text{Weight (kg)}}{\text{Height (m)}^2}.\]

BMI is a widely used indicator of body fatness and provides a general measure of whether an individual falls within a healthy weight range [6]. In addition to BMI, waist-to-hip ratio (WHR) was calculated by dividing the waist circumference by the hip circumference, which provides a measure of fat distribution and central obesity. Waist circumference (WC) was measured at the narrowest part of the torso, typically just above the iliac crest, using a flexible measuring tape. Both WHR and WC are important indicators of central obesity, which is closely associated with metabolic and cardiovascular risk factors [3].

Sleep quality was assessed using the Pittsburgh Sleep Quality Index (PSQI), a validated self-reported questionnaire that measures various aspects of sleep quality and sleep disturbances over the previous month [7]. The PSQI includes 19 items grouped into seven components: subjective sleep quality, sleep latency, sleep duration, habitual sleep efficiency, sleep disturbances, use of sleep medication, and daytime dysfunction. The total PSQI score ranges from 0 to 21, with higher scores indicating poorer sleep quality. A PSQI score above five is generally considered indicative of significant sleep disturbances [8]. The PSQI is a widely used tool in sleep research and has been validated in diverse populations, making it suitable for assessing sleep quality in young adults.

The data obtained were entered into an MS Excel sheet (Microsoft Corporation, Redmond, Washington, USA), and statistical analysis was performed. Descriptive statistics were calculated for all variables. Correlation analyses were conducted to examine the relationships between anthropometric indices and PSQI scores. Multiple linear regression analysis was performed to assess the impact of BMI, WHR, and WC on sleep disturbances while controlling for potential confounding factors such as age, gender, and lifestyle habits.

## Results

This study was conducted to examine the relationship between anthropometric indices of obesity and sleep disturbances in young adults. This study included 243 participants aged between 18 and 30 years, recruited from volunteers at Sri Lakshmi Narayana Institute of Medical Sciences. The following results were obtained from the statistical analysis done. 

Table 1 provides a summary of the demographic characteristics of the study sample, which consists of 243 participants. The mean age of the participants is 24.2 years, with a median age of 25 years, indicating that the majority of participants are young adults. The age range spans from 18 to 30 years, with a standard deviation of 3.2 years, suggesting that the ages are relatively clustered around the mean. In terms of gender distribution, the sample comprises 113 males (46.5%) and 130 females (53.5%), reflecting a slightly higher proportion of female participants.

**Table 1:** Demographic data of the study participants.

Age	
Mean age	24.2 years
Median age	25 years
Age range	18-30 years
Standard deviation	3.2 years
Gender distribution	
Male count (N (%))	113 (46.5%)
Female count (N (%))	130 (53.5%)

Table 2 provides the distribution of participants reveals that the majority are classified within the normal weight category (146 participants, 60%), while 24 participants (10%) are underweight, 49 participants (20%) are overweight, and 24 participants (10%) are obese, as per BMI. Regarding waist-to-hip ratio (WHR), 170 participants (70%) are at low risk, while 73 participants (30%) are at high risk. For waist circumference (WC), 122 participants (50%) fall into the low-risk category, with the remaining 121 participants (50%) at higher risk. In terms of sleep quality, 194 participants (80%) have poor sleep quality according to their PSQI scores, while 49 participants (20%) report good sleep quality.

**Table 2:** Descriptive statistics and percentage distribution of BMI, WHR, WC, and PSQI scores among study participants. BMI: body mass index, WHR: waist-to-hip ratio, WC: waist circumference, PSQI: Pittsburgh Sleep Quality Index.

Variable	Mean	Standard deviation	Categories	N (%)
BMI	23.6	5.0	Underweight (<18.5)	24 (10%)
			Normal weight (18.5-24.9)	146 (60%)
			Overweight (25.0-29.9)	49 (20%)
			Obese (≥30.0)	24 (10%)
WHR	0.85	0.08	Low risk (<0.85 for women, <0.90 for men)	170 (70%)
			High risk (≥0.85 for women, ≥0.90 for men)	73 (30%)
WC	82.7 cm	11.1 cm	Low risk (<94 cm for men, <80 cm for women)	122 (50%)
			High risk (≥94 cm for men, ≥80 cm for women)	121 (50%)
PSQI score	11.5	5.3	Good sleep quality (≤5)	49 (20%)
			Poor sleep quality (>5)	194 (80%)

The correlation analysis in Table 3 reveals significant positive relationships between body composition measures (BMI, WHR, and WC) and Pittsburgh Sleep Quality Index (PSQI) scores. Higher values in BMI (r = 0.28, p = 0.004), WHR (r = 0.23, p = 0.013), and WC (r = 0.30, p = 0.001) are associated with poorer sleep quality. These correlations indicate that increased body fat, especially abdominal fat, is linked to worse sleep quality.

**Table 3:** Correlation between body mass index, waist-to-hip ratio, and waist circumference with sleep quality scores. *p value < 0.05, which is considered significant. BMI: body mass index, WHR: waist-to-hip ratio, WC: waist circumference, PSQI: Pittsburgh Sleep Quality Index.

Variable pair	Pearson correlation coefficient (r)	Significance (p-value)
BMI and PSQI	0.28	0.004*
WHR and PSQI	0.23	0.013*
WC and PSQI	0.30	0.001*

The regression analysis values in Table 4 demonstrate significant associations between body mass index (BMI), waist-to-hip ratio (WHR), waist circumference (WC), and Pittsburgh Sleep Quality Index (PSQI) scores. Higher values for BMI (β = 0.31, p = 0.0007), WHR (β = 2.05, p = 0.0003), and WC (β = 0.11, p < 0.0001) are positively associated with worse PSQI scores. This suggests that increases in body fat, particularly abdominal fat, lead to poorer sleep quality.

**Table 4:** Regression analysis of BMI, WC, and WHR on PSQI scores. *p value < 0.05, which is considered significant. BMI: body mass index, WHR: waist-to-hip ratio, WC: waist circumference, PSQI: Pittsburgh Sleep Quality Index.

Predictor	Coefficient (β)	Standard error	t-value	p-value
Intercept (β0)	1.54	0.80	1.93	0.054
BMI (β1)	0.31	0.09	3.44	0.0007*
WHR (β2)	2.05	0.56	3.67	0.0003*
WC (β3)	0.11	0.03	4.04	<0.0001*

As shown in Table 5, the regression analysis for males indicates significant associations between BMI (β = 0.27, p = 0.007), WHR (β = 1.80, p = 0.011), and WC (β = 0.09, p = 0.027) with PSQI scores. Increased BMI, WHR, and WC are all linked to poorer sleep quality, highlighting the impact of higher body fat and central obesity on sleep in males.

For females, BMI (β = 0.34, p = 0.002), WHR (β = 2.20, p = 0.001), and WC (β = 0.14, p < 0.0001) are significantly associated with PSQI scores. Each unit increase in these variables corresponds to worse sleep quality, reflecting the notable effect of body composition on sleep in females.

**Table 5:** Gender-specific regression analysis of BMI, WC, and WHR on PSQI scores. *p-value < 0.05, which is considered significant. BMI: body mass index, WHR: waist-to-hip ratio, WC: waist circumference, PSQI: Pittsburgh Sleep Quality Index.

Predictor	Coefficient (β)	Standard error	t-value	p-value
Males				
Intercept (β0)	2.75	1.00	2.75	0.007*
BMI (β1)	0.27	0.10	2.70	0.007*
WHR (β2)	1.80	0.70	2.57	0.011*
WC (β3)	0.09	0.04	2.23	0.027*
Females				
Intercept (β0)	0.85	0.95	0.89	0.375
BMI (β1)	0.34	0.11	3.09	0.002*
WHR (β2)	2.20	0.64	3.43	0.001*
WC (β3)	0.14	0.03	4.67	<0.0001*

As shown in Table 6, the ANOVA test results demonstrate sleep quality across BMI categories, showing that individuals with higher BMI categories tend to experience poorer sleep quality. Specifically, underweight individuals have an average PSQI score of 11.46, and normal-weight individuals have a better average score of 10.38. Overweight individuals report a higher average score of 12.46, indicating worse sleep quality and obese individuals have the highest average score of 13.65, reflecting the poorest sleep quality.

**Table 6:** Comparison of mean PSQI scores across BMI categories. BMI: body mass index, PSQI: Pittsburgh Sleep Quality Index.

BMI category	Mean PSQI score	Standard deviation	Number of participants
Underweight	11.46	5.34	24
Normal weight	10.38	5.2	146
Overweight	12.46	5.4	49
Obese	13.65	5.6	24

## Discussion

This study examined the relationship between obesity, measured using body mass index (BMI), waist-to-hip ratio (WHR), and waist circumference (WC), and sleep disturbances, as measured by the Pittsburgh Sleep Quality Index (PSQI) among participants. The findings revealed that higher values in BMI, WHR, and WC are significantly associated with sleep disturbances. Regression analysis further confirmed that increases in BMI, WHR, and WC are significantly associated with worsening PSQI scores.

These findings highlight the detrimental impact of increased body fat and central obesity on sleep quality. The positive correlations between BMI, WHR, and WC with PSQI scores suggest that greater body fat and abdominal fat are associated with sleep disturbances. This aligns with the hypothesis that excess body fat contributes to sleep disturbances through mechanisms such as obstructive sleep apnea (OSA) and increased systemic inflammation [9].

Our results are consistent with prior research indicating a negative impact of obesity on sleep quality. For instance, Finkelstein et al. (2012) and Koenig et al. (2020) reported significant associations between high BMI and poor sleep quality, supporting our findings [10,11]. However, while our study observed a moderate correlation between BMI and sleep quality, some studies have reported stronger associations [12]. This discrepancy may be attributed to differences in study populations or methodologies. Peppé et al. (2017) found a strong link between central obesity, measured by WHR, and sleep disorders, which aligns with our findings regarding the significant impact of WHR on sleep quality [13].

The physiological effects of excess body fat explain the associations between body composition and sleep quality. Abdominal obesity, in particular, contributes to obstructive sleep apnea (OSA) by exerting pressure on the airway, leading to repeated breathing interruptions during sleep. This strong link between abdominal fat and OSA is well-documented in research by Javaheri et al. (2017), Young et al. (2002), and Gotsopoulos et al. (2002), all of which show that OSA significantly worsens sleep quality [14-16].

Visceral fat also contributes to poor sleep by promoting chronic low-grade inflammation, with elevated inflammatory markers like TNF-α and IL-6 disrupting sleep patterns. Studies by Panagiotakos et al. (2005) and Yudkin et al. (1999) support this connection between inflammation and sleep disturbances [17,18]. Additionally, obesity-related hormonal imbalances, such as altered levels of leptin and ghrelin, disrupt sleep by affecting appetite regulation and metabolic processes. Spiegel et al. (2004) found that leptin resistance contributes to sleep disturbances [19], and Taheri et al. (2004) linked short sleep duration with reduced leptin, elevated ghrelin, and increased BMI [20].

Moreover, increased body fat leads to heightened sympathetic nervous system activity, which can interfere with the ability to fall asleep and maintain restorative sleep. Bradley and Floras (2003) highlighted how this sympathetic activation, often seen in obesity, plays a significant role in sleep disturbances [21]. These physiological mechanisms are consistent with the study's findings, further validating the observed relationship between body composition and sleep quality.

## Conclusions

This study highlights a clear link between obesity measures such as BMI, WHR, and WC and sleep quality. Obesity associated with poorer sleep is likely due to factors like airway obstruction, inflammation, and hormonal imbalances. These findings align with existing research, emphasizing the negative impact of obesity on sleep. The results suggest that addressing body weight and reducing abdominal fat could improve sleep quality and reduce sleep disturbances.
